# Supplementary material for: DnaK response to expression of protein mutants is dependent on translation rate and stability
Source: Commun Biol. 2022 Jun 16;5:597. doi: 10.1038/s42003-022-03542-2 (PMC9203555; doi:10.1038/s42003-022-03542-2)
Supplement: Supplementary file 1 — Supplementary Information [file 42003_2022_3542_MOESM1_ESM.pdf]

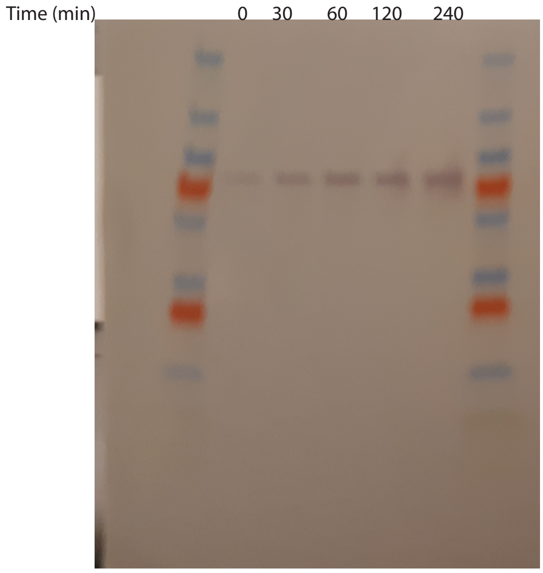

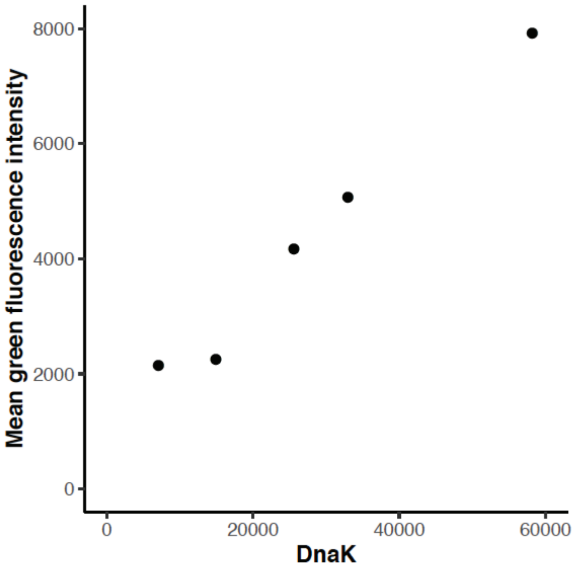

Figure S1 *From a culture expressing Q33S, samples were taken prior to induction (time = 0 min), 30 min, 60 min, 120 min and 240 min after induction. Expression of DnaK was analysed by western blot and quantified by imageJ. The quantified amount of DnaK is compared to the green fluorescence signal measured at the same time point on the culture the sample was taken from. The correlation between DnaK and green fluorescence is 0.99.*

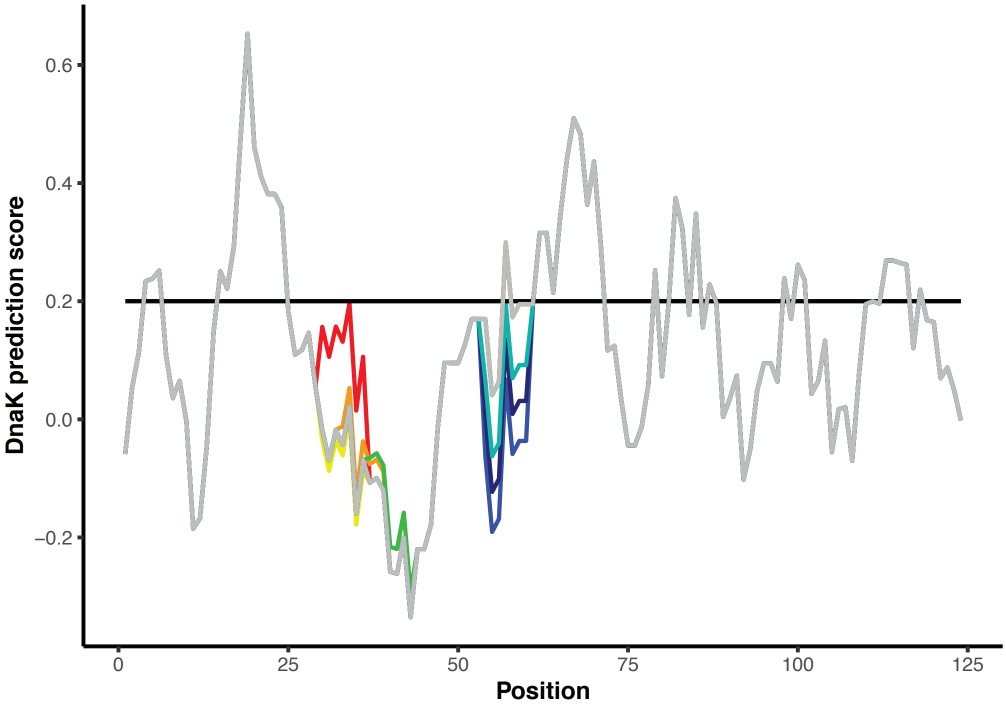

Figure S2 *DnaK binding sites predicted by the qualitative ChaperISM algorithm {Guiterres, 2020} for variants : L57G (dark blue), L57C (blue), L57P (cyan), M40A (green), Q33S (yellow), V36I (orange), Q33Y (red) and wt (grey). Where only grey is visible the score is identical for all variants. Cut-off for DnaK binding is 0.2 (black).*

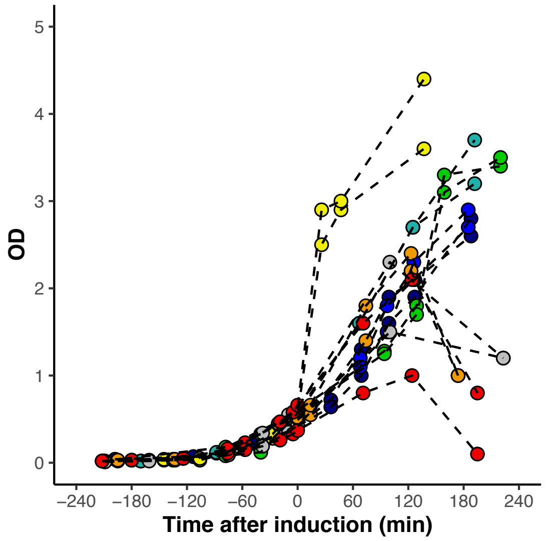

Figure S3 OD_{600} *as a function of time after induction for cells expressing the variants: L57G (dark blue), L57C (blue), L57P (cyan), M40A (green), Q33S (yellow), V36I (orange), Q33Y (red) and wt (grey). Two of three replicates are shown.*

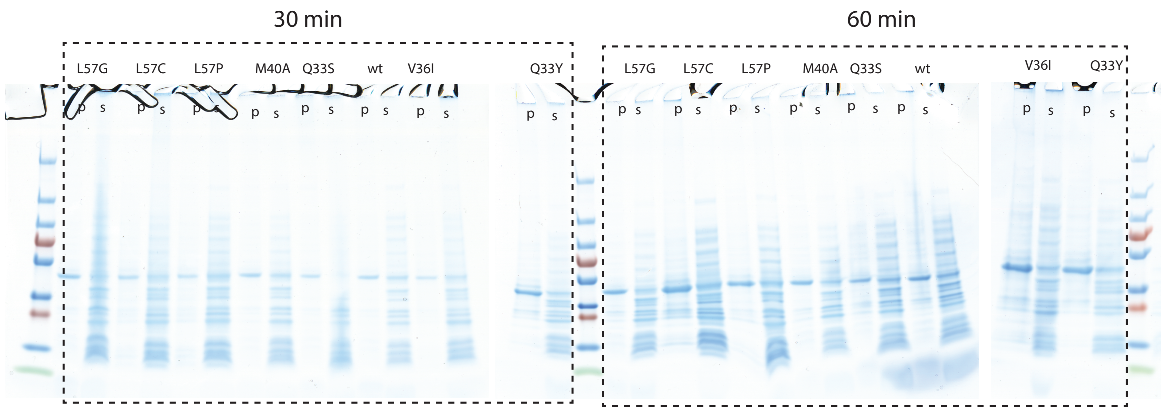

Figure S4 Samples were taken 30 and 60 min after induction of expression of the eight N102LT variants. Soluble (s) and insoluble fraction (p) was separated and analysed by SDS-PAGE.

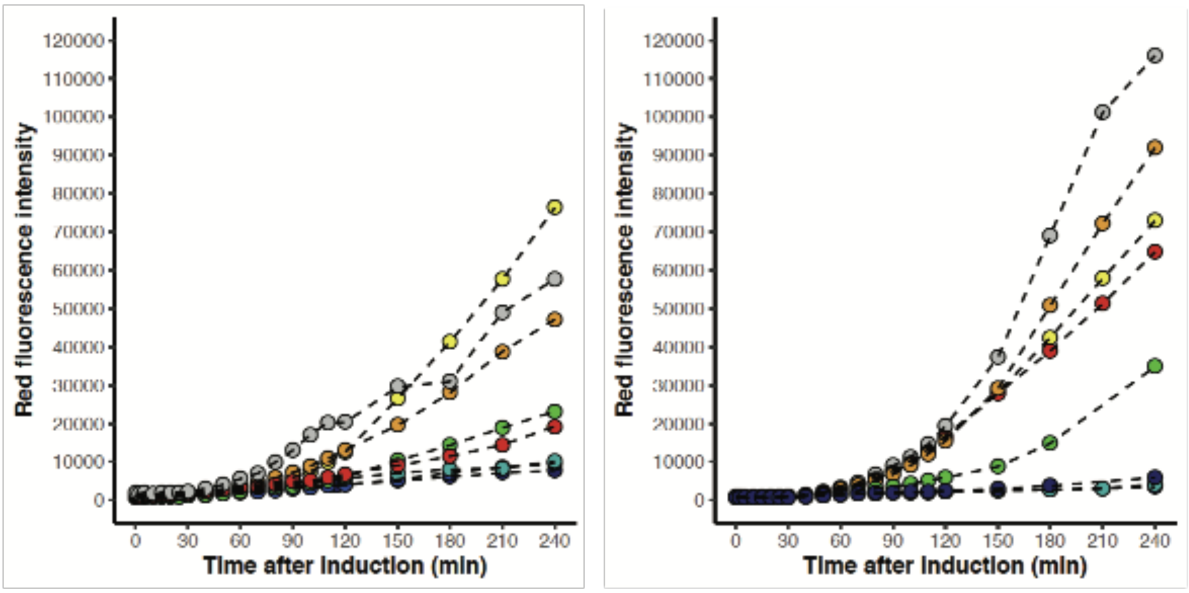

Figure S5 *Mean red fluorescence intensity of cells expressing variants: L57G (dark blue), L57C (blue), L57P (cyan), M40A (green), Q33S (yellow), V36I (orange), Q33Y (red) and wt (grey). Two replicates (of three total replicates) of each variant are shown in two separate plots.*

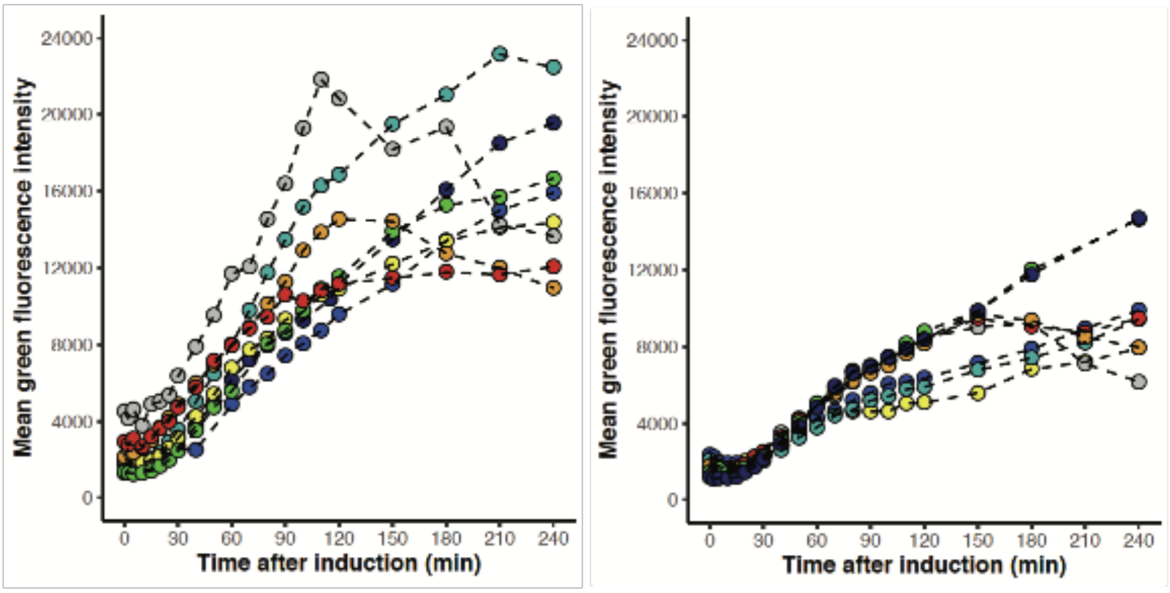

Figure S6 *Mean green fluorescence intensity of cells expressing variants: L57G (dark blue), L57C (blue), L57P (cyan), M40A (green), Q33S (yellow), V36I (orange), Q33Y (red) and wt (grey). Two replicates (of three total replicates) of each variant are shown in two separate plots.*

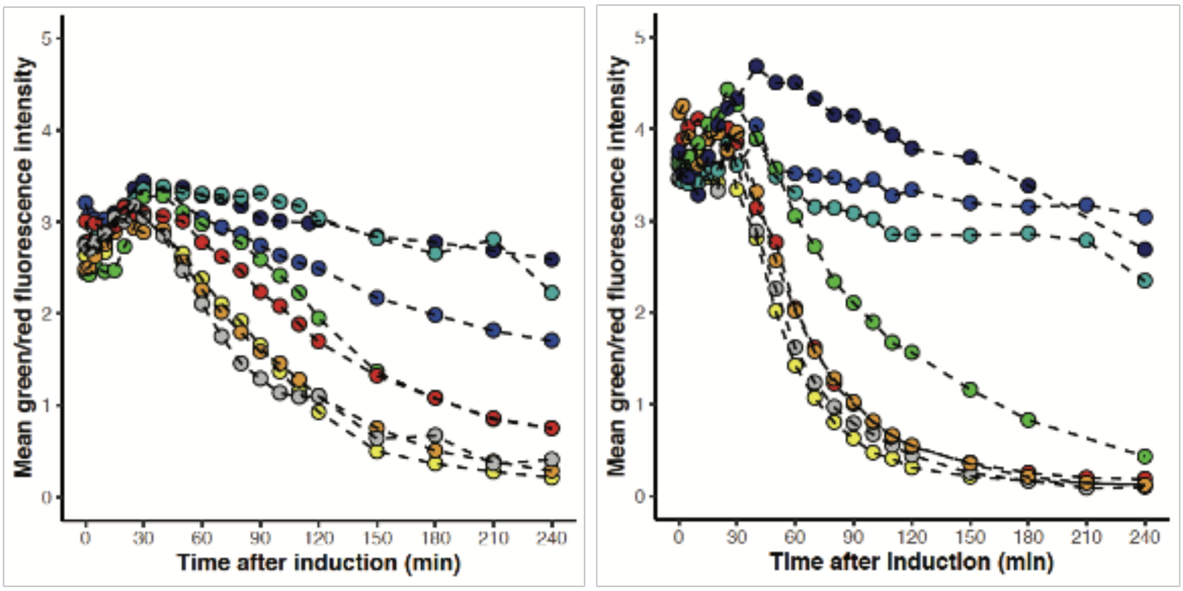

Figure S7 *Mean green/red fluorescence intensity of cells expressing variants: L57G (dark blue), L57C (blue), L57P (cyan), M40A (green), Q33S (yellow), V36I (orange), Q33Y (red) and wt (grey). Two replicates (of three total replicates) of each variant are shown in two separate plots.*

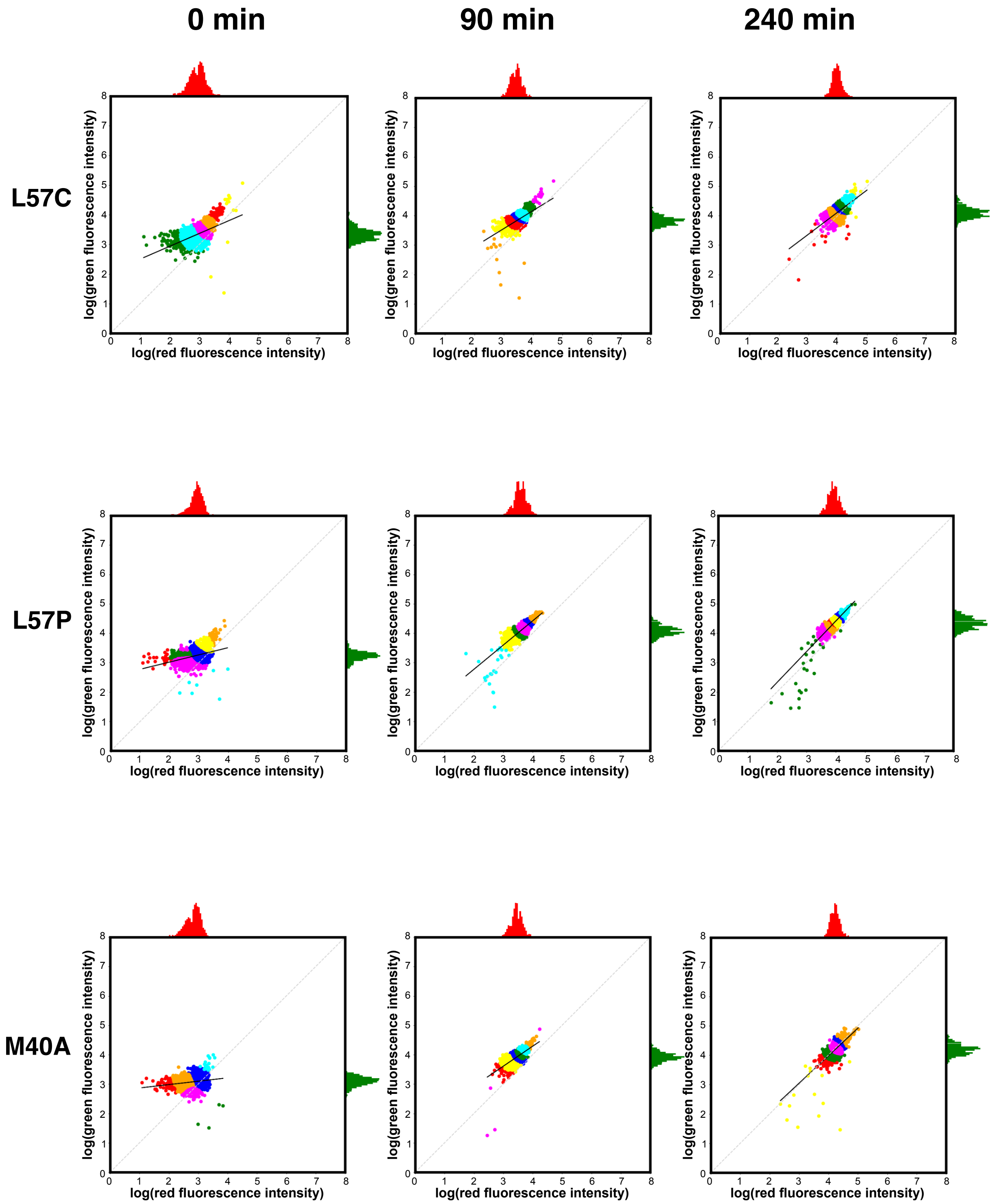

Figure S8 *The red and green fluorescence for each individual cell in a population expressing L57C (top), L57P (middle) and M40A (bottom) at the time of induction (left) and 240 min after induction with IPTG (right). Colouring is based on a Bayesian Gaussian mixture model for cluster assignment. The x-axis histogram (red) shows the distribution of red fluorescence, and the y-axis histogram (green) shows the distribution of green fluorescence within the population.*

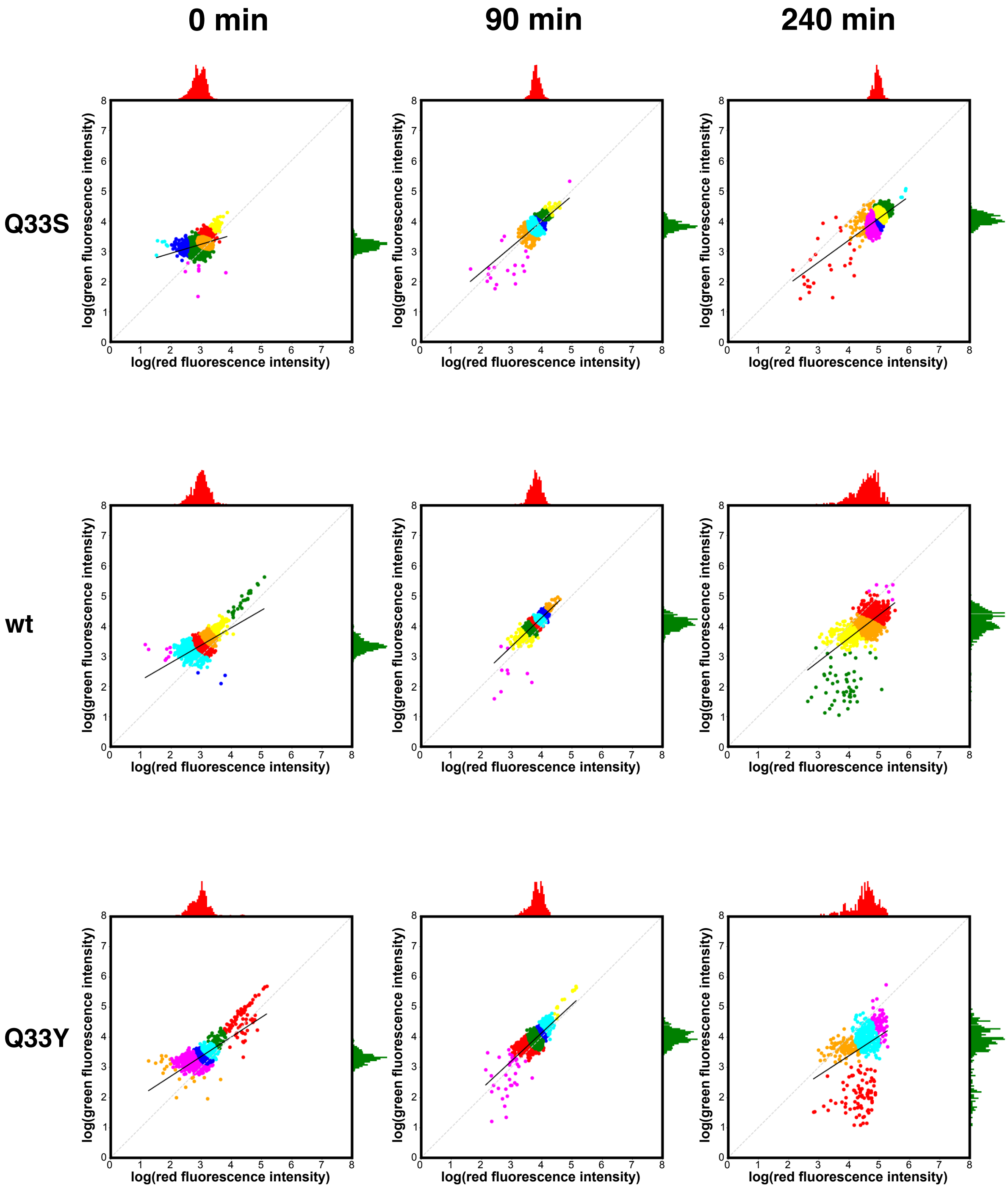

Figure S9 *The red and green fluorescence for each individual cell in a population expressing Q33S (top), wt (middle) and Q33Y (bottom) at the time of induction (left) and 240 min after induction with IPTG (right). Colouring is based on a Bayesian Gaussian mixture model for cluster assignment. The x-axis histogram (red) shows the distribution of red fluorescence, and the y-axis histogram (green) shows the distribution of green fluorescence within the population.*

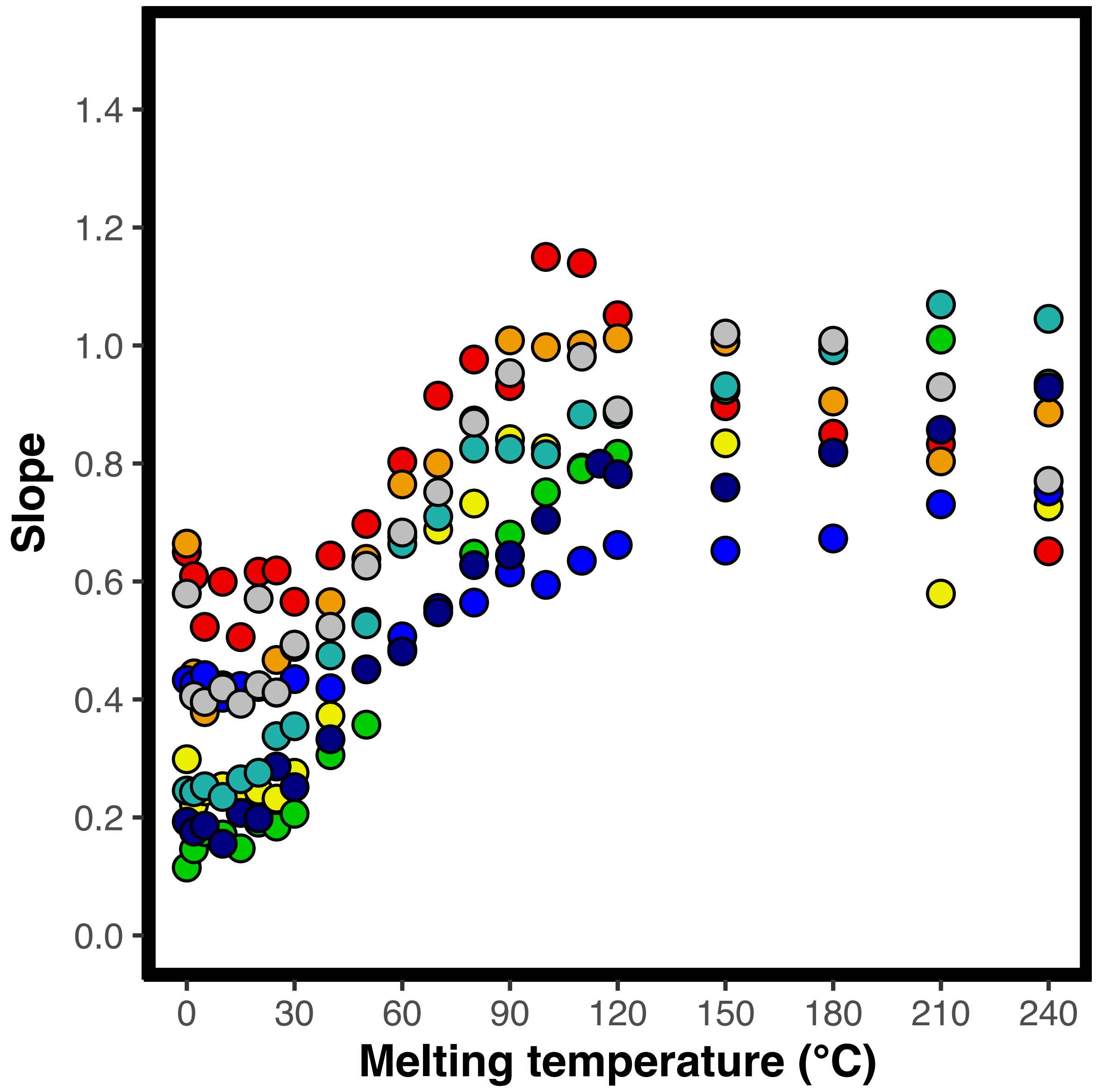

S 10 For a population of cells expressing the variants: L57G (dark blue), L57C (blue), L57P (cyan), M40A (green), Q33S (yellow), V36I (orange), Q33Y (red) and wt (grey) the slope of log transformed red fluorescence vs log transformed green fluorescence is calculated. One of three replicates are shown for each variant.

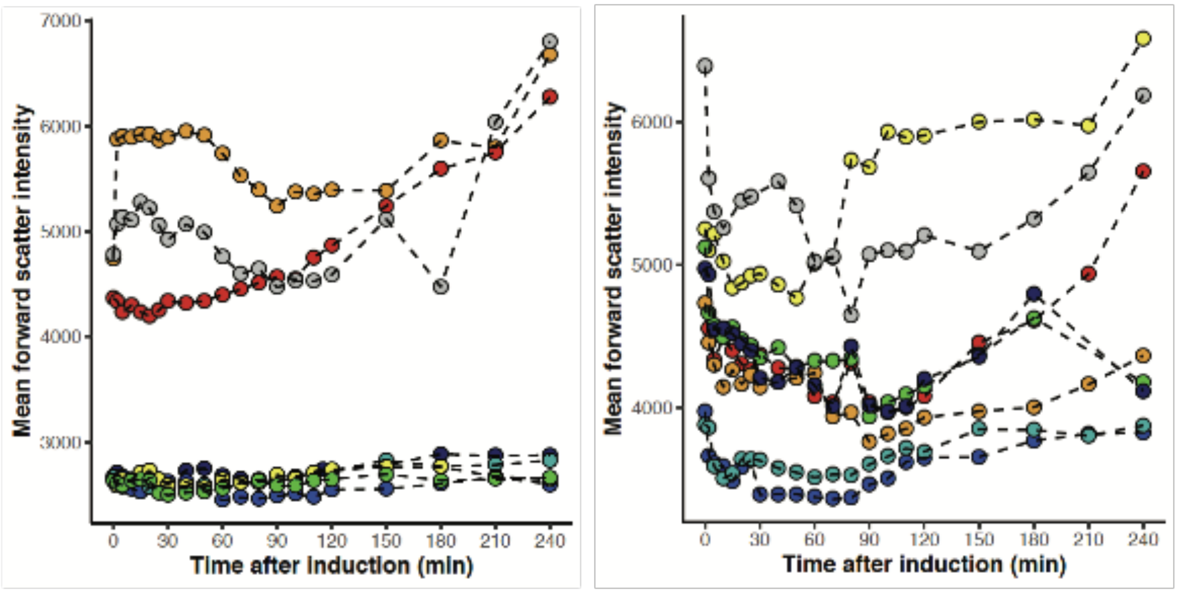

S 11 Forward scatter as a function of time for variants: L57G (dark blue), L57C (blue), L57P (cyan), M40A (green), Q33S (yellow), V36I (orange), Q33Y (red) and wt (grey). Two replicates (of three total replicates) of each variant are shown in two separate plots.

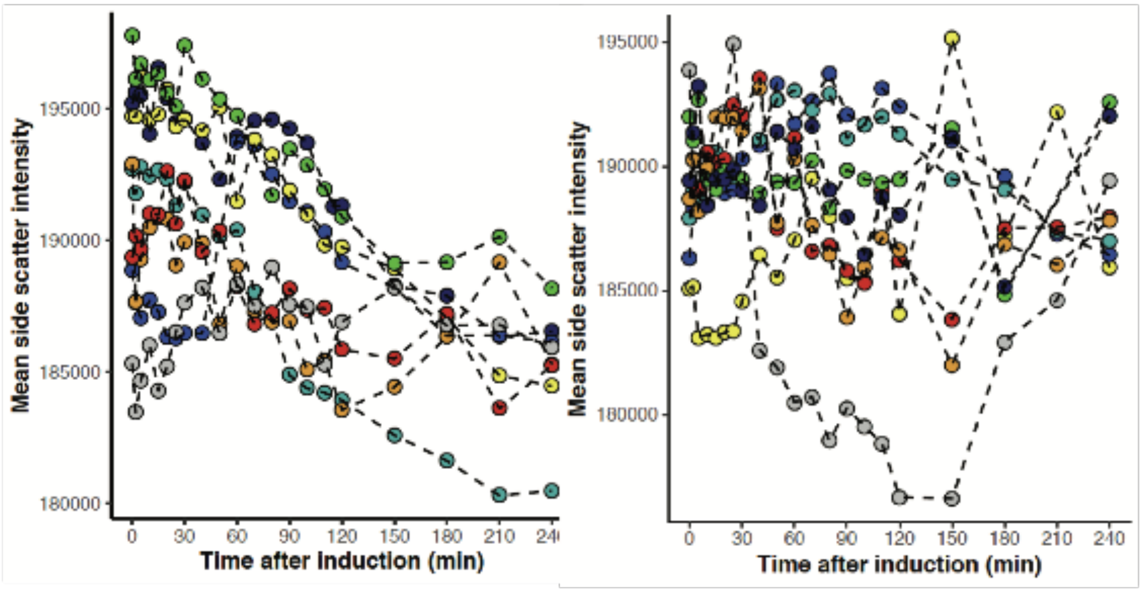

S 12 Side scatter as a function of time for variants: L57G (dark blue), L57C (blue), L57P (cyan), M40A (green), Q33S (yellow), V36I (orange), Q33Y (red) and wt (grey). Two replicates (of three total replicates) of each variant are shown in two separate plots.

Supplementary text

To estimate the effect of dilution on the fluorescence signal we developed a mathematical model to describe the kinetics of protein synthesis in a cell. The time-dependence of protein expression can be described by (see for example [1, 2])

$$\frac{dc}{dt} = k_c - \mu(t)c \qquad [1]$$

Where c is the protein concentration, k_c is the protein synthesis rate and μ the growth rate of the cell. The second term describes the effect of dilution due to cell growth. Assuming that the fluorescence is linearly dependent on concentration the time-dependence of fluorescence can be modeled as:

$$\frac{df}{dt} = k_f[k_c - \mu(t)\, c\,] \qquad [2]$$

Where f is the fluorescence and k_f is the fluorescence per protein. In principle the protein synthesis rate can also depend on the cell growth rate, but for simplicity we assume it is constant. In the simulations we set k_c in the range 0.15-5 min^{-1} based on experimentally observed values in Nordholt et al. [2]. However, the value of kc had negligible impact on the result. We fitted the time-dependent optical density data for the third replicate (which has more OD measurement points) to a Logistic model (Figure SX1) using the curveball python package[3]

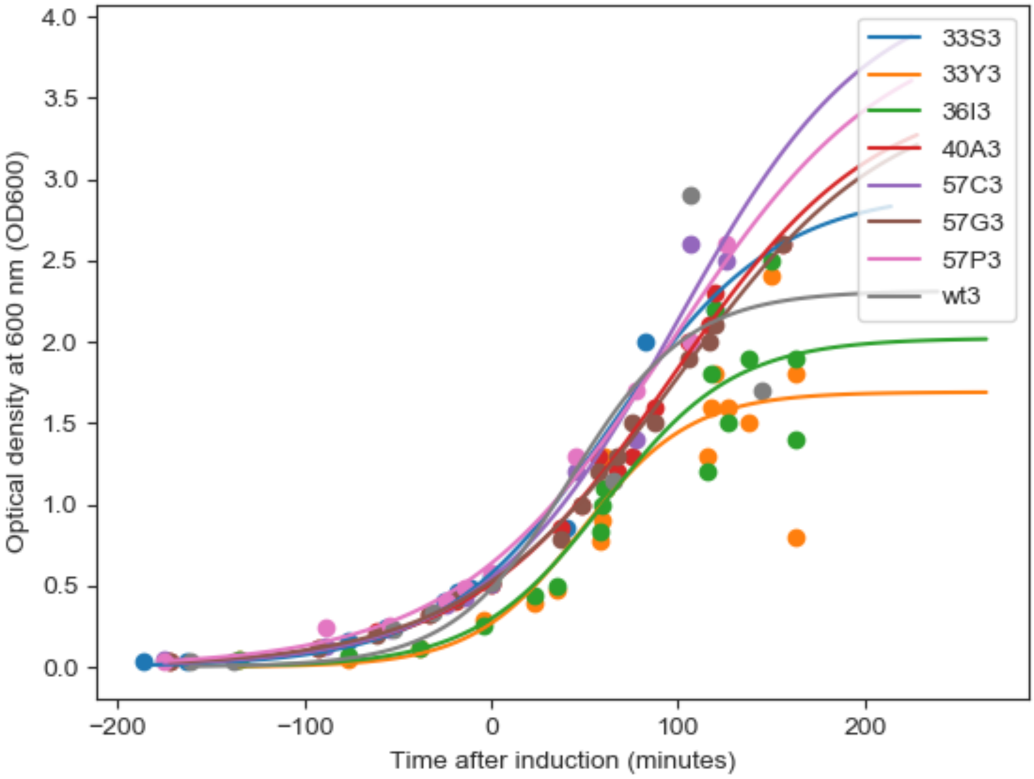

Supplementary text Figure : Fit of optical density (OD at 600 nm) as a function of time using a logistic growth model for a selected number of variants. Model fit shown as lines.

We then numerically solved the differential equation in equation 2 using the growth rates calculated using the fitted logistic growth model but using the same synthesis rate for all variants. The relative fluorescence (calculated as the experimental data) was then evaluated in the 30-60 interval minute after induction and plotted in Figure SX2.

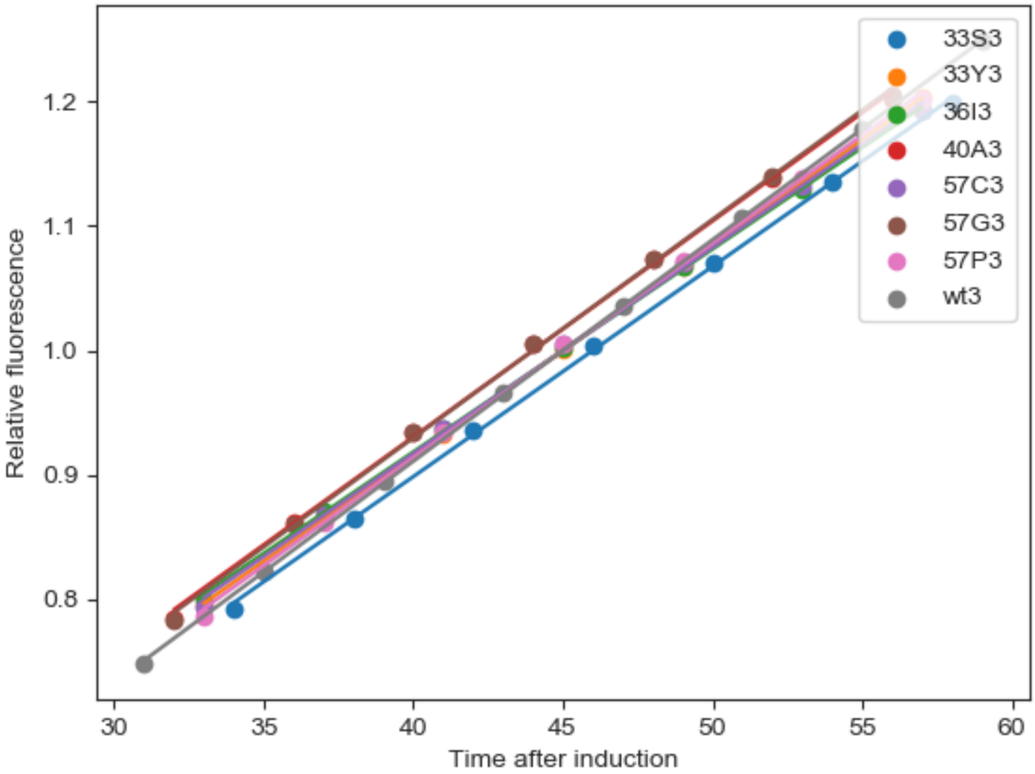

Supplementary text Figure 2: Relative fluorescence values as a function of time in the interval 30 to 60 minutes after induction for a selected number of variants. Fluorescence values are normalized with their average values within the interval. Linear fit to data shown as lines.

Linear regression was applied to extract the rate of relative fluorescence change. Since the underlying true rate is the same for all variants in this simulation, the effective rates report on the effect of dilution. Based on this analysis, the effect of growth dilution on the relative fluorescence rates is associated with a standard deviation of 2.7%. The small impact is largely due to the similar growth rate between variants in the 30-60 minutes time window. For replicate 1 and 2, 33S had a significantly different growth curve with a 30% change in relative fluorescence rate compared to the average.

The analysis of the data at the 60–90 minute window is complicated by the increasing effect of proteins degradation. The growth rates are smaller in this time window resulting in a smaller dilution effect, but this is counteracted by increased variation in cell growth rate between variants. The standard deviation in relative fluorescence is estimated to 12% for the 60-90 minute window.

Some of the predicted variation is likely a consequence of the relatively noisy OD data, suggesting that the true dilution effect is smaller.

1. Hintsche M, Klumpp S. Dilution and the theoretical description of growth-rate dependent gene expression. J Biol Eng. 2013;7(1):22. Epub 2013/09/18. doi: 10.1186/1754-1611-7-22.
2. Nordholt N, van Heerden J, Kort R, Bruggeman FJ. Effects of growth rate and promoter activity on single-cell protein expression. Sci Rep. 2017;7(1):6299. Epub 2017/07/26. doi: 10.1038/s41598-017-05871-3.
3. Ram Y, Dellus-Gur E, Bibi M, Karkare K, Obolski U, Feldman MW, et al. Predicting microbial growth in a mixed culture from growth curve data. Proc Natl Acad Sci U S A. 2019;116(29):14698-707. Epub 2019/06/30. doi: 10.1073/pnas.1902217116.
