# Supplementary material for: DnaK response to expression of protein mutants is dependent on translation rate and stability
Source: Commun Biol. 2022 Jun 16;5:597. doi: 10.1038/s42003-022-03542-2 (PMC9203555; doi:10.1038/s42003-022-03542-2)
Supplement: Supplementary file 2 — Reporting Summary [file 42003_2022_3542_MOESM2_ESM.pdf]

nature portfolio

Corresponding author(s): Ingemar Andre
Signe Christensen

Last updated by author(s): Apr 3, 2022

Reporting Summary

Nature Portfolio wishes to improve the reproducibility of the work that we publish. This form provides structure for consistency and transparency in reporting. For further information on Nature Portfolio policies, see our Editorial Policies and the Editorial Policy Checklist.

Statistics

For all statistical analyses, confirm that the following items are present in the figure legend, table legend, main text, or Methods section.

n/a	Confirmed	
☒	☐	The exact sample size (n) for each experimental group/condition, given as a discrete number and unit of measurement
☐	☒	A statement on whether measurements were taken from distinct samples or whether the same sample was measured repeatedly
☒	☐	The statistical test(s) used AND whether they are one- or two-sided<br>*Only common tests should be described solely by name; describe more complex techniques in the Methods section.*
☒	☐	A description of all covariates tested
☒	☐	A description of any assumptions or corrections, such as tests of normality and adjustment for multiple comparisons
☒	☐	A full description of the statistical parameters including central tendency (e.g. means) or other basic estimates (e.g. regression coefficient) AND variation (e.g. standard deviation) or associated estimates of uncertainty (e.g. confidence intervals)
☒	☐	For null hypothesis testing, the test statistic (e.g. F, t, r) with confidence intervals, effect sizes, degrees of freedom and P value noted<br>*Give P values as exact values whenever suitable.*
☐	☒	For Bayesian analysis, information on the choice of priors and Markov chain Monte Carlo settings
☒	☐	For hierarchical and complex designs, identification of the appropriate level for tests and full reporting of outcomes
☐	☒	Estimates of effect sizes (e.g. Cohen's d, Pearson's r), indicating how they were calculated

Our web collection on statistics for biologists contains articles on many of the points above.

Software and code

Policy information about availability of computer code

Data collection	Prosort v. 1.6 was used for collection of flow cytometry data
Data analysis	Flow cytometry data was analysed using the flowCore, flowViz and flowWorkspace packages within the Bioconductor frame work in R version 3.6.3

For manuscripts utilizing custom algorithms or software that are central to the research but not yet described in published literature, software must be made available to editors and reviewers. We strongly encourage code deposition in a community repository (e.g. GitHub). See the Nature Portfolio guidelines for submitting code & software for further information.

Data

Policy information about availability of data

All manuscripts must include a data availability statement. This statement should provide the following information, where applicable:
- Accession codes, unique identifiers, or web links for publicly available datasets
- A description of any restrictions on data availability
- For clinical datasets or third party data, please ensure that the statement adheres to our policy

Code and data are available at github (https://github.com/Andre-lab/DnaK_response_manuscript)

Field-specific reporting

Please select the one below that is the best fit for your research. If you are not sure, read the appropriate sections before making your selection.

☒ Life sciences ☐ Behavioural & social sciences ☐ Ecological, evolutionary & environmental sciences

For a reference copy of the document with all sections, see nature.com/documents/nr-reporting-summary-flat.pdf

Life sciences study design

All studies must disclose on these points even when the disclosure is negative.

Sample size	Not applicable as the study is performed with bacterial cells
Data exclusions	No data was excluded
Replication	Flow cytometry analysis was replicated twice for all eight variants.
Randomization	Not applicable as the study is performed with bacterial cells
Blinding	Not applicable as the study is performed with bacterial cells

Reporting for specific materials, systems and methods

We require information from authors about some types of materials, experimental systems and methods used in many studies. Here, indicate whether each material, system or method listed is relevant to your study. If you are not sure if a list item applies to your research, read the appropriate section before selecting a response.

Materials & experimental systems

n/a	Involved in the study	
☒	☐	Antibodies
☒	☐	Eukaryotic cell lines
☒	☐	Palaeontology and archaeology
☒	☐	Animals and other organisms
☒	☐	Human research participants
☒	☐	Clinical data
☒	☐	Dual use research of concern

Methods

n/a	Involved in the study	
☒	☐	ChIP-seq
☐	☒	Flow cytometry
☒	☐	MRI-based neuroimaging

Flow Cytometry

Plots

Confirm that:

☒ The axis labels state the marker and fluorochrome used (e.g. CD4-FITC).

☐ The axis scales are clearly visible. Include numbers along axes only for bottom left plot of group (a 'group' is an analysis of identical markers).

☐ All plots are contour plots with outliers or pseudocolor plots.

☐ A numerical value for number of cells or percentage (with statistics) is provided.

Methodology

Sample preparation	The samples analysed are E. coli BL21(de3) pLysS cells transformed with reporter plasmid harbouring the gene of interest as well as two fluorescent reporter proteins (tag-RFP and sf-GFP). Cells were grown in liquid culture and protein synthesis were induced with IPTG prior to analysis by flow cytometry.
Instrument	Biorad s3e cell sorter
Software	Data was collected using Biorads Prosort software v. 1.6. Flow cytometry data was analysed using the flowCore, flowViz and flowWorkspace packages within the Bioconductor frame work in R version 3.6.3
Cell population abundance	Samples were not sorted only analysed.

Gating strategy

Cells were gated on forward and side scatter to exclude doublets.

☐ Tick this box to confirm that a figure exemplifying the gating strategy is provided in the Supplementary Information.
